# Photobiomodulation for Photoreceptor Rescue in Retinal Disease: Mitochondrial, Redox, Vascular, and Translational Perspectives—A Narrative Review

**DOI:** 10.3390/antiox15081034

**Published:** 2026-08-19

**Authors:** Mario D. Toro, Alessandro Avitabile, Roberta Amato, Dario Rusciano, Caterina Gagliano

**Affiliations:** 1 Eye Clinic, Department of Public Health, University of Naples Federico II, 80131 Naples, Italy; mariodamiano.toro@unina.it; 2 Chair and Department of General and Paediatric Ophthalmology, Medical University of Lublin, 20-079 Lublin, Poland; 3 Department of Special Surgery, University of Jordan, Queen Rania St., Amman 11942, Jordan; 4 Biomedicine, Neuroscience and Advanced Diagnostics (BIND) Department, University of Palermo, 90128 Palermo, Italy; alessandro.avitabile@unipa.it; 5 Neurovisual Science Technology, Research Division (NEST-G.R.I.D.O.), 95123 Catania, Italy; robertaamato@nestweb.it; 6 Department of Medicine and Surgery, Kore University of Enna, 94100 Enna, Italy; caterina.gagliano@unikore.it; 7 Mediterranean Foundation G.B. Morgagni, Ophthalmology Clinic, 95125 Catania, Italy

**Keywords:** photobiomodulation, photoreceptors, oxidative stress, mitochondria, cytochrome c oxidase, red light, near-infrared light, age-related macular degeneration, retinal degeneration, diabetic retinopathy

## Abstract

Photoreceptors work in a biologically demanding compartment of the eye. They consume large amounts of energy, receive continuous light and oxygen, and renew outer-segment membranes enriched in polyunsaturated lipids. These conditions are necessary for vision, but they also make the outer retina poorly tolerant to persistent mitochondrial dysfunction and oxidative stress. Photobiomodulation (PBM), mainly based on red and near-infrared light, has been investigated as a way to support retinal cells that are functionally impaired but not yet irreversibly lost. The field has also acquired new clinical relevance after the 2024 De Novo marketing authorization by the United States Food and Drug Administration (FDA) of the Valeda Light Delivery System for dry age-related macular degeneration (AMD). This narrative review examines the mitochondrial, redox, inflammatory, and neurovascular mechanisms proposed for PBM, and discusses preclinical and clinical evidence across nonexudative AMD, inherited retinal degeneration, diabetic retinal disease, and light-induced damage. Current findings are encouraging, but devices, doses, schedules, endpoints, and sponsorship patterns differ substantially among studies. PBM therefore deserves further investigation, especially in early or intermediate disease, but its clinical use should remain linked to tested protocols, rigorous safety monitoring, and biomarkers of residual retinal functional reserve.

## 1. Introduction: Photoreceptors, Oxidative Stress, and the Rationale for Photobiomodulation

The retina is not simply neural tissue placed at the back of the eye. It is neural tissue that works in direct contact with light while maintaining one of the highest metabolic rates of the body. Photoreceptors sustain phototransduction, ionic gradients, synaptic transmission, and daily outer-segment renewal through a close metabolic exchange with the retinal pigment epithelium (RPE). This combination of energy demand, oxygen exposure, and structural turnover helps explain why oxidative injury has remained central to retinal disease research [1,2].

Photoreceptors occupy a particularly vulnerable position in this setting. Their outer segments are enriched in polyunsaturated fatty acids, especially docosahexaenoic acid, which helps preserve membrane fluidity and visual function but is also susceptible to peroxidation [1]. Their inner segments contain densely packed mitochondria, required for phototransduction, ion gradients, synaptic transmission, and continuous renewal of outer-segment discs. At the same time, photoreceptors lie near the choroidal circulation, one of the most highly perfused vascular beds in the body. Thus, photoreceptors need oxygen to survive, but this dependence on oxygen also exposes them to oxidative injury.

Reactive oxygen species (ROS) are not simply toxic by-products of metabolism. At controlled levels, they participate in redox signaling, metabolic adaptation, mitochondrial communication, and stress responses. Problems arise when their production exceeds the capacity of antioxidant and repair systems. In the outer retina, this imbalance can lead to lipid peroxidation, mitochondrial damage, protein oxidation, impaired autophagy, DNA injury, inflammatory signaling, and eventually cell death [2,3]. This does not imply that oxidative stress is the sole cause of retinal degeneration. Age-related macular degeneration (AMD), inherited retinal dystrophies, diabetic retinal disease, and light-induced retinal injury have different initiating mechanisms. Yet many of them converge, at least in part, on mitochondrial dysfunction and redox imbalance [2,3].

In this context, mitochondrial dysfunction and redox imbalance are plausible therapeutic targets even when they are not the first cause of disease. An intervention that increases the resistance of photoreceptors and adjacent cells could slow progression without correcting every upstream defect. Photobiomodulation (PBM) has attracted attention for this reason: the eye is accessible to light, and several proposed PBM effects intersect with mitochondrial metabolism, inflammation, and cell survival [4,5].

PBM should not be equated with a chemical antioxidant. Its proposed action is rather to modify energy metabolism and stress signaling in cells that remain viable. Depending on wavelength, dose, timing, and tissue state, mitochondrial and non-mitochondrial photoacceptors may influence cytochrome c oxidase (CCO) activity, nitric oxide (NO) signaling, adenosine triphosphate (ATP) production, membrane potential, calcium handling, and transcriptional responses [5,6].

This interpretation sets an important limit. PBM cannot replace photoreceptors that have already been lost. Its most defensible use would be earlier, when photoreceptors or RPE cells are impaired but still recoverable. Candidate settings include intermediate AMD, selected stages of inherited retinal degeneration, early diabetic retinal stress, and acute photo-oxidative injury. Whether these different conditions share enough responsive biology to justify similar protocols is still unresolved.

Several recent articles have considered PBM in AMD, and one recent review has framed the topic mainly through a mitochondrial bioenergetic perspective [7]. The present review intentionally takes a broader and more explicitly translational position. It considers photoreceptor rescue across several retinal disease settings, compares mitochondrial and redox mechanisms with glial, vascular, and temporal components, and discusses how ophthalmic trials should be designed so that PBM is not treated as generic red-light exposure. The novelty is therefore not the simple claim that PBM may affect mitochondria, but the attempt to place this claim within disease-specific retinal biology, clinical trial heterogeneity, regulatory developments, and the practical question of residual retinal functional reserve (Table 1).

In this review we discuss photoreceptor metabolic vulnerability, proposed PBM mechanisms, experimental and clinical evidence, and the practical problems that still prevent firm clinical positioning. Particular attention is given to dosimetry, device dependence, pulsed versus continuous exposure, safety, biomarker selection, regulatory status, and the difference between retinal PBM and other forms of light-based neuromodulation.

## 2. Literature Search Strategy

This review was developed as a narrative and mechanistic analysis rather than as a systematic review or meta-analysis. Relevant articles were identified through targeted searches of PubMed/MEDLINE, Scopus, Web of Science Core Collection, CrossRef, ClinicalTrials.gov, and publisher websites up to July 2026. To make the search strategy more reproducible, the following Web of Science Core Collection-compatible Boolean query was used as the reference query for the scoping search: (photobiomodulation OR “low-level light therapy” OR “red light” OR “near-infrared light” OR NIR) AND (retina OR retinal OR photoreceptor* OR “retinal pigment epithelium” OR RPE OR “age-related macular degeneration” OR AMD OR “diabetic retinopathy” OR “diabetic macular edema” OR “retinal degeneration” OR “light-induced retinal injury”) AND (mitochondri* OR “oxidative stress” OR “reactive oxygen species” OR ROS OR “cytochrome c oxidase” OR CCO OR “nitric oxide” OR NO). The main time window used for this scoping check was January 2016 to July 2026, with older foundational papers retained when they were needed for mechanism, metabolism, safety, or clinical context.

Search results were screened at the title and abstract level, together with reference chasing from key reviews, clinical trials, and mechanistic papers. Approximately 180 records were inspected during this process, about 90 were examined in full-text or detailed abstract form, and 64 sources were retained in the reference list because they directly supported mechanistic, preclinical, clinical, regulatory, or methodological statements made in the manuscript. Original experimental studies, clinical trials, mechanistically informative reviews, regulatory documents, and translational articles were prioritized. English-language peer-reviewed articles were included when they directly supported the claims made in the text. Conference abstracts, trial registry entries, and regulatory records were used only when they provided information not yet available in full peer-reviewed form, and were explicitly identified as such.

A formal quantitative bibliometric or VOSviewer study was not used as a selection method, because the manuscript remains a narrative review. However, the scoping search was used to orient the reader to the structure of the field. Three recurrent thematic clusters were evident: (i) mitochondrial photobiology, including CCO, NO, ATP production, ROS signaling and alternative photoacceptors; (ii) retinal oxidative injury, including photoreceptor/RPE stress, glial activation, light damage, diabetes and inherited degeneration; and (iii) clinical translation, including AMD trials, device-specific protocols, regulatory status, reporting quality, sponsorship and long-term safety. The review does not claim to provide an exhaustive or quantitatively reproducible evidence synthesis. Its purpose is to organize the mechanistic and clinical literature into a critical translational framework aligned with SANRA principles [8].

## 3. Photoreceptor Bioenergetics and Redox Vulnerability

**Figure 1:**
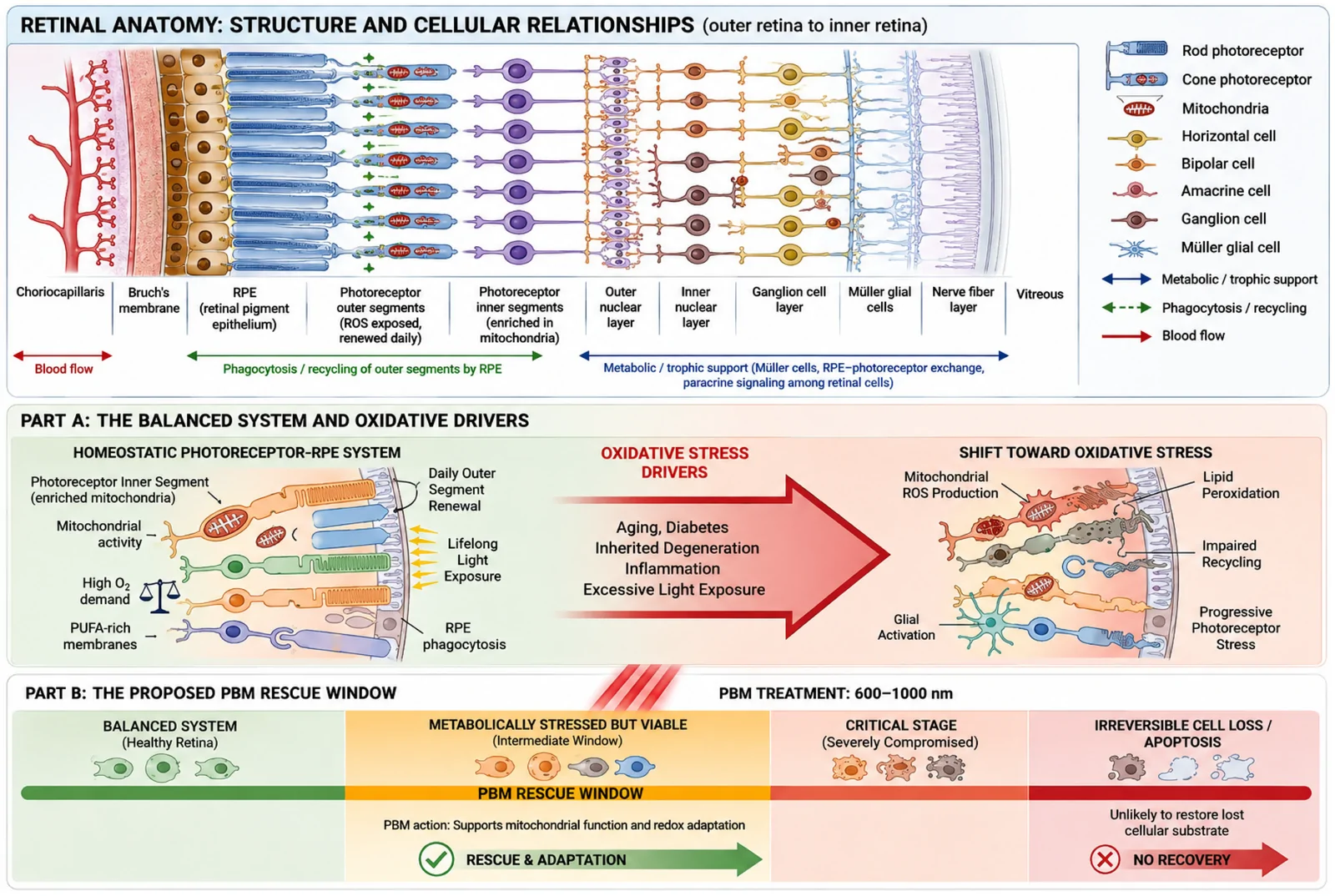
Retinal anatomical orientation, oxidative vulnerability of the photoreceptor–RPE system, and proposed PBM rescue window. The upper panel provides a simplified anatomical orientation of the retina, from the choroidal side to the vitreous side. It shows the spatial relationship among the choriocapillaris, Bruch’s membrane, retinal pigment epithelium (RPE), photoreceptor outer segments, mitochondria-rich photoreceptor inner segments, outer nuclear layer, inner nuclear layer, ganglion cell layer, Müller glial cells, nerve fiber layer, and vitreous. Horizontal, bipolar, amacrine, and ganglion cells are shown schematically to orient the reader within the retinal circuitry. Red arrows indicate blood flow, green dashed arrows indicate RPE-associated phagocytosis/recycling of photoreceptor outer segments, and blue arrows indicate metabolic/trophic support within the retinal cellular environment. This panel is intended to help readers localize the main cellular compartments discussed in the text and should not be interpreted as a complete histological representation of the retina. **Part** (**A**) summarizes the homeostatic photoreceptor–RPE system and the oxidative drivers that may shift it toward progressive stress. In the balanced state, photoreceptors combine high oxygen demand, lifelong light exposure, PUFA-rich outer-segment membranes, mitochondrial activity in the inner segment, and daily outer-segment renewal coupled to RPE phagocytosis. Aging, diabetes, inherited degeneration, inflammation, and excessive light exposure may promote mitochondrial ROS production, lipid peroxidation, impaired recycling, glial activation, and progressive photoreceptor stress. **Part** (**B**) illustrates the proposed PBM rescue window. PBM in the 600–1000 nm range is hypothesized to be most useful when retinal cells are metabolically stressed but still viable, because mitochondrial and redox adaptation may still be possible. In contrast, when degeneration has progressed to severe structural compromise, irreversible cell loss, or apoptosis, PBM is unlikely to restore the absent cellular substrate. The figure is a conceptual scheme developed by the authors and is intended to support interpretation of the review, not to imply that all retinal diseases follow identical stages or respond to the same PBM protocol. **Abbreviations:** PBM, photobiomodulation; PUFA, polyunsaturated fatty acid; RPE, retinal pigment epithelium; ROS, reactive oxygen species.

For anatomical orientation, the retina should be considered as a layered neuroepithelial tissue in which the photoreceptor–RPE–choroidal unit has a distinctive metabolic organization. From the choroidal side inward, the choriocapillaris supplies the outer retina across Bruch’s membrane; the retinal pigment epithelium contacts, supports, and phagocytoses photoreceptor outer segments; the photoreceptor inner segments contain the dense mitochondrial compartment required for energy production; and the outer nuclear layer contains photoreceptor nuclei. More internally, bipolar, horizontal, amacrine, ganglion, and Müller glial cells contribute to retinal signaling, metabolic support, and tissue homeostasis. This spatial organization is important because the oxidative and mitochondrial vulnerability discussed below does not concern isolated photoreceptors alone, but the integrated photoreceptor–RPE–choroidal environment summarized in Figure 1.

Photoreceptors are unusual neurons. They must remain electrically active in darkness, respond rapidly to light, restore ionic gradients after stimulation, maintain synaptic communication with second-order neurons, and renew their outer segments continuously. These tasks require a large and steady energy supply. Measurements of oxygen consumption in isolated mouse retina have shown that photoreceptors account for much of the retinal oxygen demand and have limited mitochondrial reserve capacity, which helps explain why they are sensitive to metabolic stress [9].

The metabolic organization of the outer retina is not a simple version of neuronal oxidative phosphorylation. Photoreceptors use glucose avidly, but a substantial fraction of this glucose is converted to lactate through a Warburg-like form of aerobic glycolysis, that is, lactate production despite the availability of oxygen [10,11]. Judged only by ATP yield, this may seem inefficient; physiologically, however, it supports the biosynthetic needs of outer-segment renewal and helps maintain the metabolic partnership between photoreceptors, Müller cells, and the RPE [10,12]. At the same time, photoreceptor mitochondria use oxidative metabolism to sustain energy-demanding functions, and fatty-acid oxidation has increasingly been recognized as part of the outer retinal metabolic landscape [11,13].

This mixed metabolic strategy gives photoreceptors flexibility but also creates vulnerability. When nutrient supply, mitochondrial respiration, lipid handling, or redox buffering becomes unstable, photoreceptors have little margin for error. Their mitochondria can become both sources and targets of oxidative stress. Electron transport chain dysfunction increases superoxide production; oxidative injury then further damages mitochondrial proteins, lipids, and mitochondrial DNA. If this cycle is not contained, bioenergetic failure and redox injury begin to reinforce each other.

The photoreceptor outer segment adds another layer of risk. Its membranes contain high levels of polyunsaturated fatty acids, which are essential for the physical properties of phototransduction membranes but also provide substrates for lipid peroxidation [1]. Daily shedding and phagocytosis of outer-segment discs by the RPE generates a heavy degradative and oxidative workload. In youth and health, this process is managed by lysosomal function, antioxidant systems, mitochondrial quality control, and efficient recycling of visual-cycle components. With aging, genetic susceptibility, diabetes, inflammation, or excessive light exposure, the same system may become less efficient. Oxidized lipids and damaged proteins can accumulate, and the RPE may shift from a supportive partner to a stressed amplifier of inflammation and degeneration [3].

Endogenous antioxidant defenses are therefore not accessory systems in the outer retina; they are central to survival. Superoxide dismutases, catalase, glutathione peroxidases, peroxiredoxins, thioredoxin-related pathways, glutathione metabolism, and NADPH-dependent reducing systems all contribute to redox balance. These defenses do not eliminate reactive oxygen species completely, and they should not. Physiological redox signaling is needed for adaptation. The problem is loss of proportion: too much oxidant pressure, too little antioxidant reserve, or a delayed repair response.

Among the transcriptional systems that regulate this balance, nuclear factor erythroid 2-related factor 2 (NRF2) has particular relevance. NRF2 controls a broad antioxidant and cytoprotective program, including genes involved in glutathione metabolism, detoxification, redox buffering, and stress resistance. In retinal models, NRF2 has been linked to protection against oxidative injury, and work in photoreceptor-like cells has shown that NRF2 deficiency can worsen blue-light-induced ROS production and cell death [14]. This is not yet direct proof in human photoreceptors in vivo. It is, however, a useful example of a broader principle: photoreceptor survival depends not only on blocking damage, but also on preserving the ability to mount an adaptive response.

Mitochondrial quality control is equally important. Damaged organelles must be repaired, diluted through biogenesis, or removed by mitophagy. Persistent defective mitochondria sustain oxidative and inflammatory signaling, whereas excessive mitophagy without compensatory biogenesis can reduce respiratory capacity. In photoreceptor-like cells, activation of mitophagy and mitochondrial stress-response pathways preserved membrane potential and improved survival during H_2_O_2_ exposure [15]. These findings support mitochondrial turnover as a relevant target, but they do not prove that PBM engages the same pathway in the human retina. The bioenergetic profile of photoreceptors therefore defines a plausible, but still provisional, PBM rescue window between reversible stress and irreversible cell loss. Figure 1 now combines a simplified anatomical orientation of the outer retina with the proposed transition from a balanced photoreceptor–RPE unit to metabolically stressed but still viable tissue.

## 4. Oxidative Stress Across Retinal Diseases

Oxidative stress appears in many retinal diseases, but it does not play the same role in all of them. Sometimes it is close to the primary injury. More often it acts as a downstream amplifier of genetic, metabolic, inflammatory, vascular, or environmental damage. Photoreceptors do not degenerate because of oxidative stress in an abstract sense. They are lost when a specific disease process pushes mitochondrial function, lipid handling, protein quality control, inflammation, or tissue repair beyond what the outer retina can still compensate.

AMD is probably the clearest example of this convergence. The macula is exposed for decades to high metabolic demand, light, oxygen, and the continuous turnover of lipid-rich photoreceptor outer segments. The RPE must phagocytose shed discs every day and process oxidized lipids, bisretinoids, damaged proteins, and complement-related signals. With aging, this work becomes less efficient. Mitochondrial injury, impaired lysosomal degradation, lipofuscin accumulation, oxidative modification of lipids and proteins, and chronic para-inflammatory signaling begin to reinforce one another [3,16]. The photoreceptor is not isolated from this process. It depends on the RPE for nutrient exchange, retinoid recycling, outer-segment clearance, and trophic support. Once the RPE becomes chronically stressed, photoreceptor survival is compromised even when the initial injury does not begin inside the photoreceptor itself.

Inherited retinal degenerations show a different pattern. In retinitis pigmentosa and related disorders, the initiating event is often a mutation affecting rods, phototransduction, ciliary transport, the visual cycle, or structural proteins. Yet the subsequent loss of cones, which is mainly responsible for the severe reduction in central and daylight vision, may involve secondary mechanisms that are not simply a direct expression of the original mutation. After rods are lost, oxygen consumption in the outer retina decreases, and the remaining cones may be exposed to a relatively hyperoxic environment. Experimental work in models of retinitis pigmentosa has shown that antioxidant treatment can reduce cone cell death [17], and that increased expression of antioxidant enzymes such as catalase and mitochondrial superoxide dismutase can protect cones [18]. These studies are important because they show that oxidative injury is not only a theoretical consequence of degeneration. In some experimental settings, modifying it can change photoreceptor survival.

Diabetic retinal disease adds another perspective. It has traditionally been described as a microvascular complication, and the vascular component remains central. However, the diabetic retina is also a metabolically stressed neural tissue. Hyperglycemia drives mitochondrial superoxide production, advanced glycation, polyol-pathway activation, protein kinase C signaling, inflammation, and vascular dysfunction [19]. These pathways injure endothelial cells, pericytes, Müller cells, neurons, and photoreceptors as part of a broader neurovascular disorder. Here oxidative stress does not act alone, but it links metabolic excess to inflammatory and vascular damage. PBM, if it improves mitochondrial handling of stress, may be most useful before advanced neurovascular disruption becomes irreversible. It should not be presented as a substitute for metabolic control, anti-vascular endothelial growth factor (anti-VEGF) therapy, laser treatment, or other established interventions when these are clinically indicated.

Light-induced retinal injury is more directly connected to the central topic of this review because it illustrates both sides of retinal light biology. Light is necessary for vision, and controlled light exposure may activate protective or adaptive pathways. Yet excessive or inappropriate exposure can damage the retina through photochemical mechanisms involving rhodopsin activation, bisretinoid phototoxicity, mitochondrial injury, lipid peroxidation, calcium imbalance, and inflammatory activation [20]. This duality must remain central in any discussion of retinal PBM. The therapeutic claim is not that any light exposure is protective. Rather, the claim is that defined light parameters may produce adaptive effects, whereas other exposures may be ineffective or harmful. The retina is therefore not only an attractive target for PBM, but also the tissue in which PBM must be most carefully dosed.

Glial cells help determine whether oxidative injury remains limited or becomes self-amplifying. Retinal microglia are not passive bystanders. They survey the retinal environment, respond to damaged neurons and RPE cells, and can shift toward phenotypes that are protective, phagocytic, inflammatory, or damaging, depending on the context [21]. Müller cells also provide metabolic and antioxidant support, but they can enter reactive states when the tissue is chronically stressed. Once glial activation becomes sustained, inflammatory mediators, nitric oxide, complement-related signals, and altered metabolic exchange may worsen photoreceptor injury. This is one reason why retinal degeneration can continue even after the initial trigger has stabilized or disappeared.

Oxidative stress is therefore not equivalent across retinal diseases. In AMD, it is interwoven with RPE dysfunction, lipid oxidation, complement activation, and para-inflammation. In inherited degeneration, it may contribute to secondary cone loss after rod death. In diabetes, it links metabolic overload to neural and vascular injury. In light damage, it participates directly in photochemical toxicity. Grouping all these conditions under a single antioxidant rationale would obscure differences in timing, cellular targets, and treatment needs.

## 5. Principles of Photobiomodulation: Light Parameters, Dose, and Safety

Photobiomodulation is sometimes described too casually as low-level light therapy. The wording is understandable historically, but it is not precise enough for retinal disease. PBM refers to the use of non-ionizing light, usually delivered by lasers or light-emitting diodes, to trigger photophysical and photochemical events in cells without causing thermal damage [22]. The purpose is not to heat tissue, ablate tissue, or create a destructive photochemical reaction. The aim is to use light as a biological signal.

Several parameters determine whether that signal is useful. Wavelength is the most obvious. Red and near-infrared light are commonly used because they penetrate tissue more effectively than shorter wavelengths and because they overlap with absorption bands of mitochondrial chromophores, especially CCO [4,5,6,23]. In the retina, however, penetration is not the only issue. Light must pass through the ocular media, reach highly specialized neural tissue, and interact with cells that are themselves designed to detect photons. This makes retinal PBM different from PBM applied to skin, muscle, or peripheral nerves. A wavelength that is safe and effective in one tissue cannot simply be assumed to behave in the same way in the retina.

Irradiance, fluence, exposure duration, treatment frequency, beam geometry, and total number of sessions are just as important as wavelength. Irradiance describes the power delivered per unit area, whereas fluence describes the energy delivered per unit area over time. A short exposure at relatively high irradiance and a longer exposure at lower irradiance may deliver a similar total energy, but the biological effects may not be identical. Cells respond not only to how much energy they receive, but also to the rate and temporal pattern of delivery. For retinal applications, this distinction is not a minor technical detail; it may determine the difference between adaptation and injury [23,24].

PBM also follows a biphasic dose–response pattern. Too little light may produce no meaningful biological effect, while excessive exposure may suppress beneficial responses or become harmful [24]. This biphasic behavior is one reason why PBM studies can be difficult to interpret. A negative result does not necessarily mean that the biological concept is wrong. It may mean that the wavelength, dose, treatment schedule, endpoint, or patient population was poorly matched to the therapeutic target. Conversely, a positive result obtained with one device or protocol cannot automatically be generalized to another. PBM is not a single intervention. It is a family of interventions defined by optical and biological parameters.

This caution is particularly important in retinal disease, because the retina already has well-characterized mechanisms of phototoxicity. Laser and optical-radiation standards such as IEC 60825-1 and ANSI Z136.1 provide general frameworks for classification and safe use of laser products [25,26], but clinical retinal PBM requires an additional ophthalmic layer of safety assessment. Blue and short-wavelength visible light can promote photochemical injury under certain conditions, especially when exposure is intense, prolonged, or combined with pre-existing susceptibility [20]. Even red-light interventions deserve caution: a JAMA Ophthalmology case report described retinal damage after repeated low-level red-light laser exposure for pediatric myopia control, a different indication and population but a useful warning that non-invasive light exposure is not automatically risk-free [27].

Red and near-infrared PBM is conceptually different from phototoxic exposure, but it must still be evaluated with the same discipline that applies to any intervention directed at the eye. Safety cannot be inferred from the non-invasive nature of the treatment. It has to be demonstrated through retinal imaging, functional testing, careful monitoring of disease progression, adverse-event reporting, and long-term follow-up.

Continuous and pulsed delivery add another layer of complexity. In classical PBM, pulsing is usually discussed in relation to tissue response, heat dissipation, mitochondrial signaling, or possible resonance with biological rhythms. In retinal PBM, pulsed exposure raises additional questions because the retina and visual pathways can respond to temporal patterns of light. This does not imply that pulsed PBM should be confused with visual flicker stimulation or gamma entrainment, but it does mean that timing may become a relevant experimental variable. A retina exposed to continuous red light, slowly pulsed near-infrared light, or frequency-structured visual stimulation may not interpret these inputs in the same way.

Retinal PBM studies should report wavelength, bandwidth, irradiance, fluence, spot size, exposure duration, number of sessions, treatment interval, beam geometry, pupil conditions when relevant, ocular media status, and retinal safety monitoring. Without these data, studies cannot be reproduced or compared, and an apparent treatment failure cannot be separated from a poorly specified protocol. Mitochondrial biology provides the rationale; optical precision and safety determine whether that rationale can be tested credibly.

## 6. Mitochondrial, Redox, and Vascular Mechanisms of Photobiomodulation

The strongest biological rationale for retinal photobiomodulation comes from mitochondria. This does not mean that every effect of PBM can be reduced to one enzyme or one pathway. The retina is too complex for that. Still, the mitochondrial hypothesis remains central because red and near-infrared light interact with a tissue whose survival depends heavily on oxidative metabolism, redox balance, and efficient stress adaptation. In photoreceptors, even modest disturbances in mitochondrial performance can influence ion gradients, synaptic activity, outer-segment renewal, and cell survival.

CCO has long been considered one of the main candidate photoacceptors for PBM. It is the terminal enzyme of the mitochondrial electron transport chain and contains heme and copper centers with absorption properties in the red to near-infrared region. Early experimental work in primary neurons showed that red and near-infrared light could partially restore CCO activity and ATP content after toxic inhibition, with the most effective wavelengths overlapping the absorption spectrum of oxidized CCO [28]. This finding does not prove that CCO explains all PBM effects, but it provides a plausible biochemical entry point for understanding how light may influence mitochondrial function.

One widely discussed mechanism is the interaction between CCO and NO. NO can bind to CCO and interfere with oxygen reduction, especially under conditions of cellular stress. According to this model, photon absorption may favor NO dissociation from CCO, improving electron transport, mitochondrial membrane potential, oxygen utilization, and ATP production [29]. Once dissociated, NO may also participate in local signaling that influences vascular tone, cellular communication, and stress responses. This idea is attractive because it connects mitochondrial respiration with local perfusion and redox signaling. It is also biologically credible in the retina, where oxygen supply, mitochondrial activity, and vascular regulation are tightly linked.

The CCO–NO model remains influential, but the strength of evidence should not be overstated. It is supported by absorption spectra, cellular responses outside the retina, and experiments using respiratory inhibitors; however, these data do not prove that CCO is the sole or dominant photoacceptor in every retinal protocol [30]. Reported effects at wavelengths with weak CCO absorption, together with proposals involving ion channels, membrane properties, calcium signaling, vascular responses, and mitochondrial-bound or interfacial water, argue against a single-mechanism account. The point is important for ophthalmology because much of the mechanistic discussion is still extrapolated from neurons, fibroblasts, muscle, or isolated mitochondria rather than from intact human retina.

ROS occupy an ambiguous place in this discussion. In conventional antioxidant thinking, ROS are often treated as harmful molecules that should be suppressed. PBM suggests a more nuanced interpretation. A small and transient ROS signal may contribute to adaptive responses, whereas persistent or excessive ROS production promotes injury. This distinction is especially relevant in photoreceptors, where ROS participate in normal signaling but become damaging when mitochondrial dysfunction, lipid peroxidation, inflammation, and impaired repair mechanisms accumulate. PBM may therefore act less by eliminating ROS than by helping cells recover a more physiological redox balance [6,29].

Downstream of these early events, several transcriptional and metabolic responses may be engaged. These include modulation of NF-κB-related inflammatory signaling, changes in anti-apoptotic and pro-survival pathways, upregulation of antioxidant enzymes, and activation of cytoprotective programs. NRF2 is particularly relevant in the retina because it regulates glutathione metabolism, detoxification enzymes, and broader antioxidant defenses, and previous experimental work has linked NRF2 activity to protection of photoreceptor cells under photo-oxidative stress [14]. PBM can be viewed as a stimulus that may help restore adaptive stress signaling rather than simply adding an external antioxidant.

In aged mice, brief 670 nm exposure increased RPE mitochondrial membrane polarization and reduced macrophage accumulation, TNF-α, and complement-related signals [31]. This experiment is useful because it shows biological responsiveness in an aged outer retina. It does not establish reversal of aging, nor does it predict a clinical effect in AMD. Species, pigmentation, ocular optics, dose, and disease stage all complicate translation.

A vascular component should also be considered. A recent pilot study in intermediate AMD reported that PBM was associated with improved choriocapillaris perfusion, reduced drusen volume, and outer retinal remodeling over short-term follow-up [32]. These data do not prove a causal hemodynamic mechanism, and the study design remains exploratory. Nevertheless, they support the view that PBM may influence the retinal–RPE–choroidal unit as a whole, not only isolated photoreceptor mitochondria.

The existence of alternative mechanisms is also illustrated by the proposal that mitochondrial-bound or interfacial water, rather than CCO alone, may contribute to near-infrared PBM effects [33]. This hypothesis remains controversial, but it is useful because it prevents reduction of retinal PBM to a single photoacceptor.

Dose and biological context limit any rescue effect. Insufficient exposure may be inactive, excessive exposure may add stress, and advanced cell loss removes the target. PBM would also be inadequate as the only treatment when infection, uncontrolled systemic disease, exudation, or another dominant process requires established therapy.

**Figure 2:**
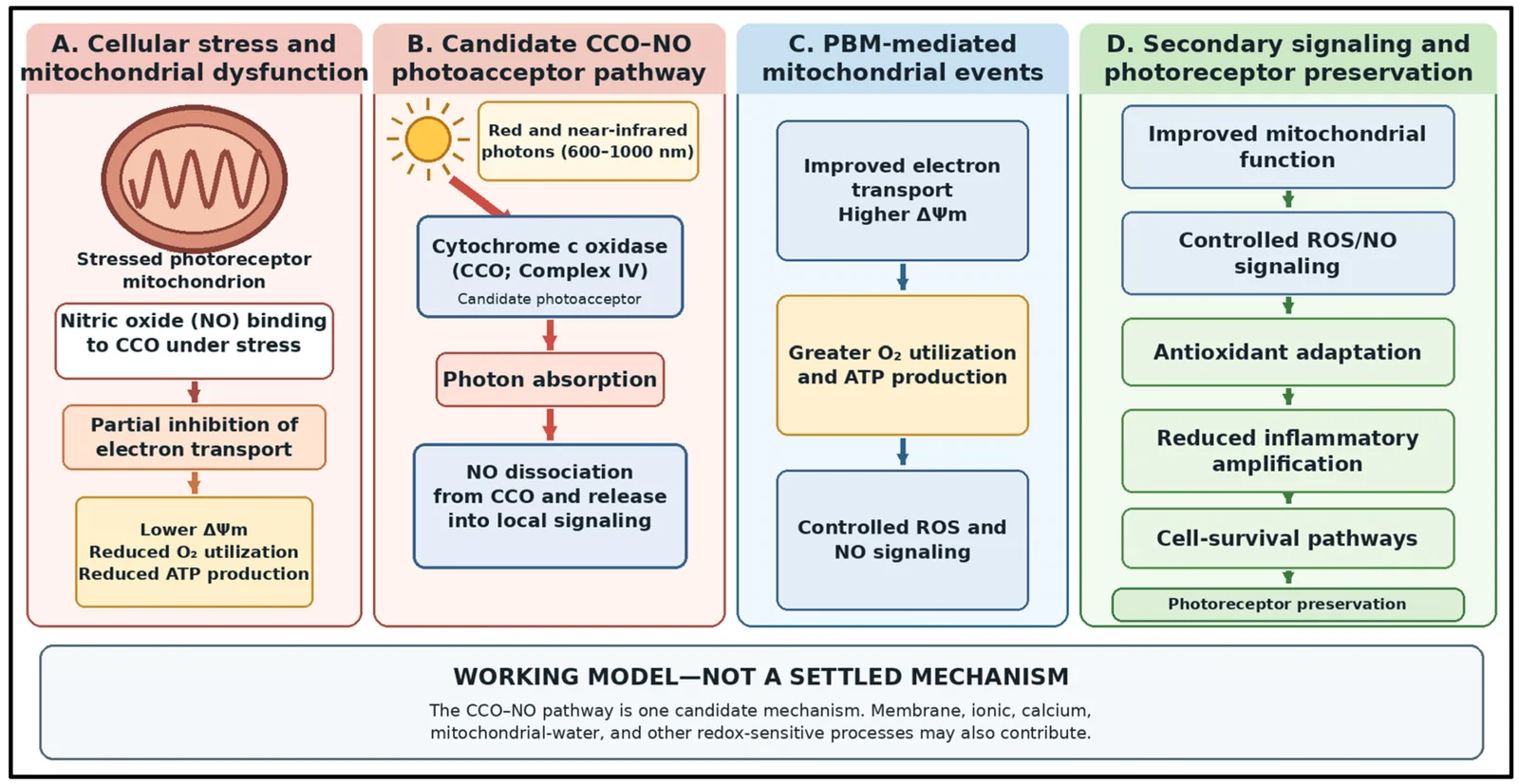
Proposed mitochondrial entry points of PBM in photoreceptor rescue. The scheme presents the CCO–NO pathway as a working model rather than a settled mechanism. Under cellular stress, NO may bind to CCO and compete with oxygen. Photon absorption may favor NO dissociation from CCO and release into local signaling, with possible recovery of electron transport, mitochondrial membrane potential, oxygen utilization, and ATP production. Controlled ROS/NO signaling may then support antioxidant adaptation, reduce inflammatory amplification, and engage cell-survival pathways. Membrane, ionic, calcium, mitochondrial-water, and other redox-sensitive mechanisms may also contribute, depending on wavelength, dose, tissue state, and disease context. Abbreviations: ATP, adenosine triphosphate; CCO, cytochrome c oxidase; NO, nitric oxide; PBM, photobiomodulation; ROS, reactive oxygen species; ΔΨm, mitochondrial membrane potential. This is a conceptual scheme developed by the authors and was not adapted from a specific published figure.

The working model is therefore one of mitochondrial and redox conditioning rather than correction of a single defect. Improved electron transport, controlled ROS signaling, antioxidant adaptation, reduced inflammatory amplification, and possibly improved choriocapillaris perfusion could slow the transition from stress to cell death, but each step remains protocol- and tissue-dependent. Figure 2 summarizes the proposed sequence; Table 2 separates mechanistic claims from useful readouts and major cautions.

## 7. Preclinical Evidence for Photoreceptor Rescue

Retinal PBM has produced protective signals in several experimental systems, but the literature is small, heterogeneous, and dominated by positive studies. Differences in species, pigmentation, injury model, wavelength, irradiance, treatment schedule, and endpoints prevent a quantitative comparison. Publication bias is also difficult to exclude.

An early study used methanol-derived formate, which inhibits CCO and causes retinal and optic-nerve toxicity. In rats, 670 nm LED treatment improved rod- and cone-mediated electroretinographic responses and reduced structural damage [34]. The model offered mechanistic proof of principle, but methanol poisoning is unlike chronic retinal degeneration and may favor an intervention directed at respiratory inhibition.

In albino rats exposed to damaging bright light, near-infrared PBM reduced photoreceptor degeneration even when administered after the insult [35]. This model is relevant to photo-oxidative injury but has important limitations: albino retinas are unusually light-sensitive, the insult is acute, and laboratory exposure bears little resemblance to the slower biology of human AMD or inherited disease.

In a related photo-oxidative model, 670 nm light reduced Müller-cell-associated microglial and macrophage activation and altered inflammatory gene expression [36]. The result supports an effect on the retinal environment rather than on photoreceptors alone. It does not resolve whether glial modulation is primary or secondary to reduced neuronal injury.

Primary murine photoreceptors exposed to blue light were protected by both 670 and 810 nm illumination, and respiratory-chain complexes I and II appeared to contribute alongside complex IV [37]. This finding challenges an exclusively CCO-based mechanism, although an isolated-cell preparation cannot reproduce ocular optics, RPE–photoreceptor exchange, choroidal perfusion, or systemic influences.

Inherited retinal degeneration models are especially relevant because they approach the clinical problem of progressive photoreceptor loss. In the P23H transgenic rat, a model of retinitis pigmentosa, 830 nm PBM preserved mitochondrial redox state, retinal function, and retinal morphology [38]. The study is important because it connected PBM with retinal mitochondrial redox status rather than only with downstream anatomy or electrophysiology. However, it remains one model, one protocol, and one disease mechanism.

Oxygen-related injury has also been explored. In C57BL/6J mice exposed to oxygen-induced retinal degeneration, 670 nm light mitigated damage and reduced signs of neural and vascular injury [39]. This model is not equivalent to human diabetic retinopathy or AMD, but it is relevant because oxygen stress, vascular changes, and neural injury appear together.

Diabetic retinal disease offers a more chronic metabolic context. In streptozotocin-diabetic mice, long-term PBM inhibited structural and functional lesions associated with diabetic retinopathy [40]. The result is encouraging because diabetes is a sustained systemic insult, but translation to patients must consider glycemic control, blood pressure, renal disease, disease duration, and established vascular damage.

Taken together, the experiments show biological plausibility across mitochondrial toxicity, photo-oxidative injury, inherited degeneration, hyperoxia, inflammation, and diabetes. They do not identify one optimal protocol, nor do they prove that the same mechanism operates in each model. Few studies directly compare wavelengths or doses, replication by independent groups is limited, and null results are probably underrepresented. Another limitation is the absence of non-human primate models or large-eye models that more closely reproduce human ocular optics, retinal dimensions, and macular anatomy.

Timing is likely to matter. Acute models may respond to early intervention before secondary inflammation develops, whereas chronic degeneration may require repeated exposure. Advanced loss is unlikely to respond because the cellular target is absent. These hypotheses require direct testing rather than inference from cross-study comparisons.

## 8. Clinical and Translational Evidence in Retinal Disease

Clinical evidence is concentrated in nonexudative AMD. Data for inherited retinal degeneration, diabetic retinopathy, and other retinal diseases remain insufficient for clinical conclusions. Dry AMD became the main testing ground because it is common, slowly progressive, and associated with mitochondrial and oxidative stress. These features make it a rational target, but they do not by themselves establish efficacy.

An early prospective study reported improvements in visual acuity, contrast sensitivity, and drusen-related measures after multiwavelength PBM [41]. Its small sample, limited control conditions, and exploratory endpoints made the findings hypothesis-generating rather than confirmatory.

LIGHTSITE I was a double-masked, randomized, sham-controlled, single-center study that reported favorable short-term functional and anatomical signals [42]. LIGHTSITE II extended the protocol to a multicenter population with intermediate nonexudative AMD [43]. Both studies improved standardization, but their sample sizes, retreatment schedules, and follow-up were still insufficient to establish durable disease modification.

LIGHTSITE III evaluated repeated courses of 590, 660, and 850 nm treatment with the Valeda system and reported improved 13-month clinical and anatomical outcomes [44]. A subsequent 24-month analysis reported that the trial met its prespecified best-corrected visual acuity (BCVA) endpoint at Month 21, with a favorable safety profile and no signs of phototoxicity [45]. These data represent the strongest clinical evidence available for retinal PBM. They also have to be read carefully. The visibility and sensory experience of treatment may complicate masking; repeated testing can influence visual-acuity outcomes; and device-specific findings cannot be generalized to other light sources.

Regulatory status has changed the clinical context. In November 2024, the United States Food and Drug Administration (FDA) granted de novo marketing authorization to the Valeda Light Delivery System as a light-based device for dry AMD [46]. This is an authorization to market a device, not a drug approval, and it should not be confused with a general endorsement of all PBM protocols or devices. The distinction is important: PBM evidence remains linked to specific devices, wavelengths, dose schedules, populations, and endpoints. Open-label extension data from LIGHTSITE IIIB are informative for long-term follow-up but remain less definitive than masked randomized evidence [47].

Recent evidence also broadens the clinical discussion. A study in early-stage dry AMD reported improvements in several visual-function outcomes after multiwavelength PBM [48]. This is relevant because earlier disease may preserve more mitochondrial and structural reserve than later intermediate or atrophic stages. At the same time, diabetic macular edema (DME) has not shown a comparable clinical signal: in Protocol AE, PBM did not produce greater improvement in central subfield thickness or visual acuity than placebo in eyes with center-involved DME and good vision [49]. A recent overview of PBM in retinal disease similarly emphasized both the promise in nonexudative AMD and the need for caution in other retinal indications [50].

PBM has not been shown to replace anti-VEGF therapy in neovascular AMD, complement inhibition in eligible geographic atrophy, or established management of diabetic macular edema and proliferative retinopathy. Its plausible role is adjunctive and stage-specific, directed toward residual cellular function rather than exudation or advanced atrophy. Commentary has appropriately highlighted durability, retreatment, endpoint interpretation, trial design, and post-market surveillance as unresolved issues [51].

Industry involvement also deserves explicit mention. Most of the strongest clinical evidence, including LIGHTSITE I-III and extension studies, is concentrated around one commercial platform and has substantial industry involvement. This does not invalidate the findings, but it increases the need for independent replication, transparent analysis, and real-world registries. A positive regulatory decision does not remove the scientific need to define who responds, how durable the response is, and how safety should be monitored during repeated treatment.

The main clinical and regulatory evidence is summarized in Table 3.

## 9. Temporal Patterning: A Limited Comparator, Not an Extension of Retinal PBM

Temporal patterning is retained in this review only as a limited comparator. The retina is not only a target for photon absorption; it is also a temporal sensory tissue that adapts to luminance, contrast, rhythm, and repeated stimulation. For this reason, the gamma-stimulation literature is useful as a cautionary example showing that patterned light can act through neural and glial timing mechanisms, not only through mitochondrial photoacceptors [52,53,54,55,56,57].

This comparison should not be overextended. Retinal PBM, as discussed here, is mainly a redox- and mitochondria-oriented intervention using red or near-infrared wavelengths, whereas 40 Hz sensory stimulation is a frequency-entrainment approach developed for neural-network modulation. The two approaches have different aims, endpoints, safety issues, and biological assumptions. The relevance for retinal PBM is therefore methodological: future studies should treat pulsed delivery, time of day, luminance, contrast, fixation status, and disease stage as protocol variables rather than as minor device details.

Accordingly, the present review does not propose that 40 Hz flicker should be incorporated into retinal PBM protocols. Any rhythmic-light strategy for retinal disease would require its own retinal safety evaluation and disease-specific mechanistic testing. This shortened section is intended to preserve the distinction between photochemical PBM and temporal neuromodulation while explaining why delivery pattern remains an experimental variable.

## 10. Clinical Translation, Reporting, and Combination Strategies

Clinical translation requires a responsive disease stage, complete optical reporting, outcomes matched to the proposed mechanism, and long-term safety assessment. These requirements are more important than broad claims about red or near-infrared light.

The most plausible candidates are eyes with measurable dysfunction but preserved outer-retinal tissue. In intermediate AMD, the aim would be to delay functional or structural decline before advanced atrophy. In inherited degeneration, the goal would be preservation rather than regeneration. In diabetes, PBM should be studied, if at all, as an adjunct during early neuroretinal stress rather than as an alternative to metabolic control or treatment of established vascular disease.

Outcome selection should reflect the disease and expected effect. BCVA alone may miss early change. Contrast sensitivity, low-luminance acuity, dark adaptation, microperimetry, electroretinography (ERG), reading performance, and patient-reported vision can complement optical coherence tomography (OCT)-based measures of drusen, ellipsoid-zone integrity, outer nuclear layer thickness, hyperreflective foci, RPE change, and fundus autofluorescence (FAF). Optical coherence tomography angiography (OCTA) may be useful where microvascular effects are hypothesized, but its role is not yet defined.

A protocol should specify the surviving cellular target, the disease process to be modified, the rationale for wavelength and dose, retreatment frequency, and the endpoint expected to change. Otherwise, PBM is tested as an exposure, not as a treatment.

Trial design should integrate three domains: optical parameters; biological measures of oxidative, mitochondrial, and inflammatory status; and ophthalmic measures of structure, function, and patient experience. This integration can distinguish a true biological response from testing variability and may reveal why apparently similar patients respond differently.

Table 4 summarizes the optical heterogeneity of representative preclinical and clinical PBM protocols discussed in the review. Values are reported only at the level needed for cross-study orientation; readers should consult the original papers for complete irradiance, fluence, beam-geometry and exposure details.

Table 5 summarizes the minimum reporting and interpretation parameters for retinal PBM studies.

Combining PBM with antioxidants or nutraceuticals is biologically plausible but unproven. AREDS and AREDS2 support supplementation in selected AMD populations, not synergy with PBM [58,59]. A factorial or otherwise adequately controlled combination trial would be needed to determine whether the approaches are additive, redundant, or even antagonistic.

The same caution applies to pharmacological combinations. Anti-VEGF agents address leakage and neovascular activity, while complement inhibitors such as pegcetacoplan and avacincaptad pegol target complement-mediated progression in selected forms of geographic atrophy [60,61]. A combination study should state whether PBM is expected to alter visual function, structural decline, treatment burden, inflammatory markers, or tissue tolerance. Vague claims of complementary action are not sufficient.

Existing PBM reporting recommendations emphasize that dose and beam parameters should be described precisely [62]. For retinal studies, it is also useful to specify whether the source is laser- or LED-based, because coherence, spectral bandwidth, beam geometry, and retinal exposure are not identical across systems [63]. Clinical trials of PBM should also be reported with the transparency expected for non-pharmacological and device-based interventions, including credible sham controls, operator details, adherence, and deviations from protocol [64].

**Figure 3:**
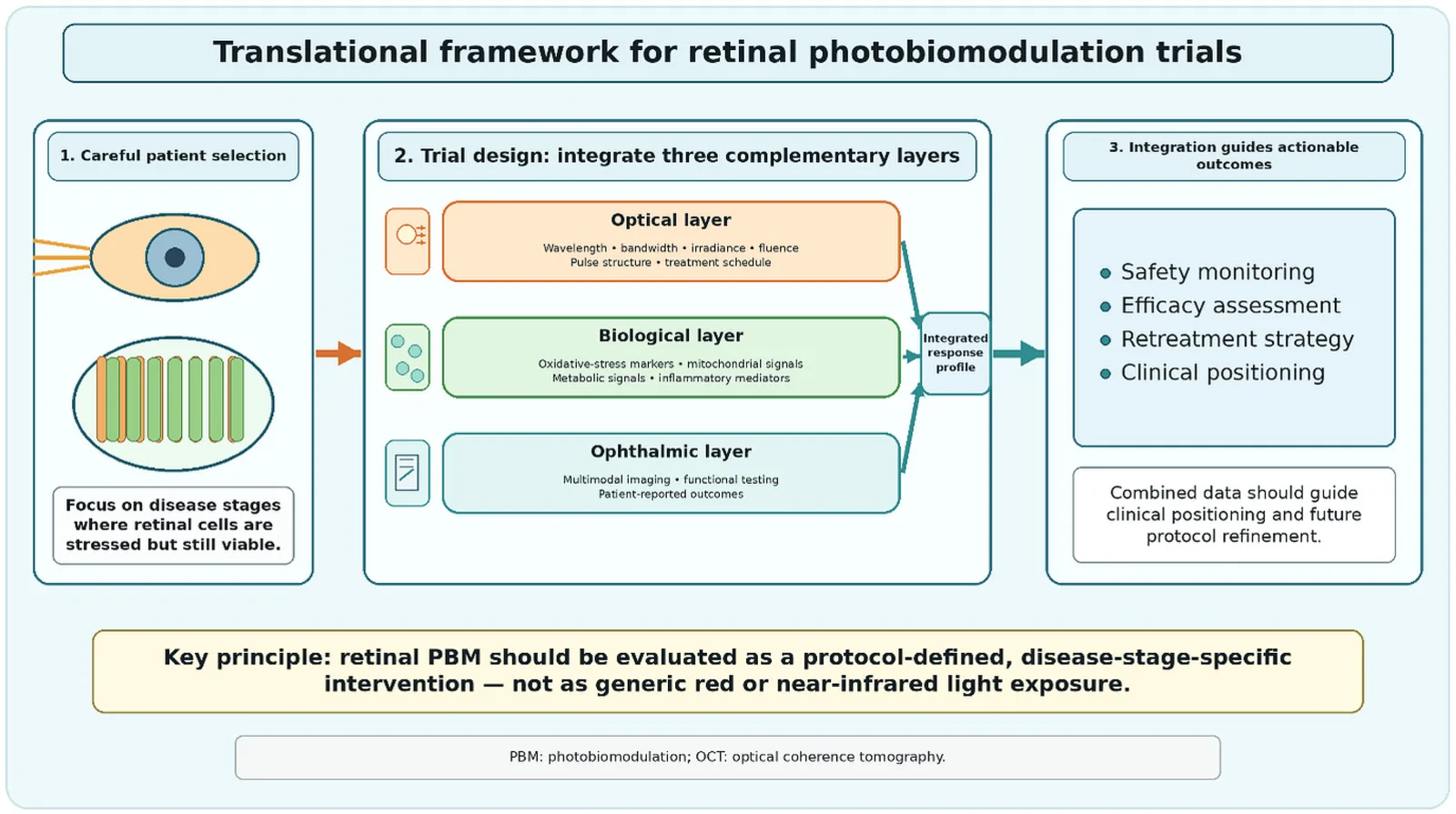
Translational framework for retinal PBM trials. The first step is careful patient selection, with emphasis on disease stages in which retinal cells are impaired but still viable. The central part shows three complementary layers that should be integrated in trial design: the optical layer, including wavelength, bandwidth, irradiance, fluence, pulse structure, and treatment schedule; the biological layer, including oxidative-stress markers, mitochondrial or metabolic signals, and inflammatory mediators; and the ophthalmic layer, including multimodal imaging, functional testing, and patient-reported outcomes. The right part indicates how these combined data should guide safety monitoring, efficacy assessment, retreatment strategy, and clinical positioning. The key message is that retinal PBM should be evaluated as a protocol-defined and disease-stage-specific intervention, not as generic exposure to red or near-infrared light. This is a conceptual scheme developed by the authors and was not adapted from a specific published figure.

Figure 3 summarizes a trial pathway based on four requirements: viable target tissue, a fully specified optical protocol, disease-appropriate outcomes, and safety monitoring comparable to that used for other ophthalmic devices.

## 11. Limitations and Research Priorities

The field is limited less by lack of plausibility than by uneven evidence. Protocols vary widely, many reports omit essential optical parameters, and outcome measures range from molecular assays to visual acuity without a common hierarchy. As a consequence, positive studies are difficult to compare and negative studies are difficult to interpret.

This review has its own methodological limitation. It is a narrative review, not a systematic review or meta-analysis. The literature search was targeted and mechanistically oriented, and it did not include formal risk-of-bias scoring, pooled effect estimation, or a PRISMA-style selection flow. For this reason, the review should be read as a critical translational synthesis aligned with SANRA principles, not as a quantitative estimate of efficacy [8].

Disease heterogeneity is equally important. AMD, inherited retinal degeneration, diabetic retinal disease, and acute light injury share some stress pathways but differ in initiating events, time course, cellular targets, and residual tissue. Cross-disease extrapolation should therefore be treated as a hypothesis, not as evidence of a universal retinal effect.

Mechanistic uncertainty remains substantial. The CCO–NO model is coherent, but direct retinal evidence is incomplete and alternative explanations involving complexes I and II, ion channels, membranes, calcium signaling, mitochondrial dynamics, vascular responses, and interfacial water have not been excluded [30,32,33]. Demonstrating a downstream change in ATP or ROS does not identify the primary photoacceptor.

Clinical trials face additional methodological problems. Visible illumination and treatment rituals complicate masking. Repeated visual testing introduces learning and regression-to-the-mean effects. Slowly progressive diseases require long follow-up, yet many studies are short and use surrogate anatomical outcomes. Independent image analysis, credible sham exposure, preregistered endpoints, transparent handling of missing data, and reporting of all adverse events are essential. CONSORT principles for non-pharmacological interventions are particularly relevant here [64].

Durability and cumulative safety are unresolved. Short-term functional improvement does not establish slower degeneration, and retreatment schedules cannot be inferred from one treatment cycle. This issue becomes critical if PBM is proposed for years of outpatient or home use.

The concentration of the strongest clinical evidence around one commercial platform and sponsor is another formal limitation of the evidence base. It does not negate the results, but it makes independent replication and registry-based post-market surveillance particularly important after regulatory authorization. Likewise, preclinical translation would benefit from large-eye models or non-human primate studies, because rodent models do not reproduce human macular anatomy or retinal optical geometry.

The main priorities are straightforward: complete protocol reporting; direct dose and wavelength comparisons; independent replication; disease-stage stratification; large-eye or primate translational models where justified; long follow-up; and combined functional, structural, and mechanistic outcomes. Null findings and adverse effects should also be published, because a literature composed mainly of positive small studies will overestimate benefit.

Temporal patterning deserves controlled experiments, not casual addition to clinical protocols. Pulsed and continuous exposure should be compared at matched energy, and time-of-day effects should be tested prospectively rather than inferred from unrelated studies.

The decisive question is whether a reproducible, clinically meaningful, and durable effect can be demonstrated for a defined retinal population and a fully specified device. Biological activity alone is not enough.

## 12. Conclusions

PBM has a credible biological rationale in retinal disease, particularly where mitochondrial dysfunction, oxidative stress, inflammation, and possibly choriocapillaris dysfunction coexist in still-viable tissue. The strongest clinical signal remains in early and intermediate nonexudative AMD, whereas evidence in other retinal disorders is still insufficient or negative, as illustrated by Protocol AE in DME. The 2024 FDA De Novo marketing authorization of the Valeda Light Delivery System has moved retinal PBM into a real clinical and regulatory setting, but it does not justify extrapolation beyond tested devices, schedules and populations. Future progress will depend less on repeating broad claims about red or near-infrared light than on transparent optical reporting, credible sham controls, independent long-term trials, post-market surveillance, disease-specific endpoints and biomarkers of residual cellular function. PBM should therefore be viewed as a protocol-defined investigational adjunct, not as a general treatment for retinal degeneration.

## Figures and Tables

**Table 1:** Positioning of the present narrative review in relation to recent PBM/AMD reviews.

Aspect	Recent PBM/AMD Reviews	Present Narrative Review
Disease scope	Usually centered on nonexudative AMD and the clinical development of PBM in that indication.	Uses AMD as the strongest clinical example, but also discusses inherited retinal degeneration, diabetic retinal disease and light-induced injury.
Mechanistic focus	Emphasis on mitochondrial bioenergetics, CCO, NO and redox signaling.	Keeps the mitochondrial–redox axis central, but adds glial activation, RPE–photoreceptor support, choriocapillaris/vascular effects, and limits of the CCO-only model.
Clinical translation	Often focused on Valeda/LIGHTSITE trials and AMD outcomes.	Adds device/protocol specificity, FDA De Novo marketing authorization, DME negative evidence, sponsor concentration, reporting standards and post-market questions.
Conceptual contribution	Useful disease-specific summaries of PBM evidence in AMD.	A cross-disease framework for asking when retinal cells are still viable enough for a PBM response and how future trials should report optical, biological and ophthalmic endpoints together.

**Table 2:** Mechanistic layers of retinal photobiomodulation, representative readouts, and interpretation cautions.

Mechanistic Focus	Core Biological Idea	Useful Readouts	Translational Caution
Mitochondrial photoacceptors/CCO–NO axis	Red/NIR photons may improve electron transport, NO signaling, membrane potential and ATP production [28,29,30].	ΔΨm; ATP; oxygen use; CCO activity; ERG; contrast sensitivity.	Working model, not a complete mechanism. Avoid a CCO-only interpretation.
Redox conditioning/antioxidant response	A brief ROS signal may activate adaptive antioxidant pathways, including NRF2-related responses [6,14,29].	ROS; lipid peroxidation; glutathione enzymes; SOD/catalase; NRF2 targets.	Likely biphasic: too little may be ineffective, too much may add stress [23,24].
Mitochondrial quality control and metabolic reserve	PBM may support mitochondrial repair, mitophagy-related adaptation, respiratory reserve and survival of stressed but viable cells [15].	Mitophagy markers; mitochondrial morphology; respiratory reserve; ONL thickness; ellipsoid zone; ERG.	Most relevant before irreversible photoreceptor or RPE loss.
Neuroinflammation and RPE–photoreceptor support	Reduced mitochondrial/redox stress may dampen Müller-cell, microglial, macrophage, complement and RPE stress signals [3,5,16,21,31].	GFAP; IBA1; TNF-α; IL-6; complement markers; hyperreflective foci; drusen/RPE measures; FAF.	Adjunctive rationale only; not a substitute for disease-specific therapies.
Vascular and choriocapillaris effects	PBM may influence the retinal–RPE–choroidal unit, including choriocapillaris perfusion and outer retinal remodeling [32].	OCTA flow deficits; drusen volume; LLVA; BCVA; outer retinal OCT markers.	Exploratory human evidence; causality and durability remain uncertain.
Dose, timing and temporal delivery	Wavelength, irradiance, fluence, pulse structure, session interval and timing may shape the response [23,24].	Complete device reporting; pulsed vs. continuous exposure; treatment schedule; safety monitoring.	PBM should be reported as a protocol, not as generic red-light exposure.

Abbreviations: ATP, adenosine triphosphate; BCVA, best-corrected visual acuity; CCO, cytochrome c oxidase; ERG, electroretinography; FAF, fundus autofluorescence; GFAP, glial fibrillary acidic protein; IBA1, ionized calcium-binding adaptor molecule 1; IL-6, interleukin 6; LLVA, low-luminance visual acuity; NIR, near-infrared; NO, nitric oxide; NRF2, nuclear factor erythroid 2-related factor 2; OCT, optical coherence tomography; OCTA, optical coherence tomography angiography; ONL, outer nuclear layer; PBM, photobiomodulation; RPE, retinal pigment epithelium; ROS, reactive oxygen species; SOD, superoxide dismutase; TNF-α, tumor necrosis factor alpha.

**Table 3:** Main clinical and regulatory evidence relevant to retinal PBM translation, including sample size, follow-up, endpoint information and sponsorship.

Study/Source	N and Design	PBM Protocol/Optical Data	Follow-Up and Primary Endpoint	Main Signal/Effect Size	Sponsorship/Limitation
Merry et al. [41]	42 eyes; exploratory dry AMD study.	LED multiwavelength PBM: 590, 670 and 790 nm; 3-week treatment course.	3 weeks and 3 months; BCVA, contrast sensitivity and drusen measures.	BCVA improved by about 5.90 letters at 3 weeks and 5.14 letters at 3 months; drusen volume decreased by 0.024 mm^3^.	Exploratory, limited control conditions; industry affiliation present.
LIGHTSITE I [42]	30 subjects/46 eyes; double-masked, randomized, sham-controlled, single-center study.	Valeda multiwavelength PBM; two treatment series, 3 times/week for 3–4 weeks over 1 year.	1 year; safety and visual/anatomical outcomes.	PBM-treated eyes showed a mean BCVA gain of 4 letters immediately after each treatment series; high responders gained 8 letters after the initial treatment.	Small trial; Valeda platform; industry involvement.
LIGHTSITE II [43]	44 subjects/53 eyes; randomized multicenter trial.	Valeda 590, 660 and 850 nm; 9 treatments/series at baseline, 4 and 8 months.	10 months; BCVA and anatomical/QoL measures.	At 9 months, PBM-treated eyes showed about 4-letter BCVA gain in full-series analysis; no phototoxicity signal.	COVID-19 disrupted protocol; Valeda platform; industry involvement.
LIGHTSITE III [44,45]	100 subjects/148 eyes; randomized controlled multicenter trial.	Valeda 590, 660 and 850 nm; 9 sessions over 3–5 weeks every 4 months.	13 and 24 months; prespecified BCVA endpoint.	13-month between-group BCVA difference about 2.4 letters; 24-month analysis reported +6.2 letters after PBM at Month 21.	Strongest clinical evidence but device-specific; masking, sponsorship, durability and disease modification remain debated.
FDA De Novo authorization [46]	Regulatory source; not a clinical trial.	Valeda Light Delivery System for dry AMD.	Decision date November 2024.	Established a regulated US marketing pathway for one light-based device.	Device-specific marketing authorization, not class-wide validation of PBM.
LIGHTSITE IIIB [47]	36 subjects/63 eyes; open-label, prospective multicenter extension; conference abstract/interim source.	Continuation or resumption of Valeda PBM.	Long-term extension follow-up.	Suggested maintained or recovered visual gains after renewed treatment following a prolonged interruption from the parent trial.	Open-label, non-randomized extension; peer-reviewed full trial report still needed.
Early-stage dry AMD study [48]	27 participants/41 eyes; retrospective clinical evaluation.	Valeda 590, 660 and 850 nm; 3 times/week over 3–5 weeks every 4 months.	Up to 16 months; VA, contrast sensitivity and ERG.	Improved multiple visual-function measures; no phototoxicity signal reported.	Single-center real-world evidence; one author employed by LumiThera.
Protocol AE in DME [49]	135 adults; phase 2 randomized trial.	670 nm PBM eye patch versus low-power white-light placebo; 90 s twice daily for 4 months.	4 months; change in OCT central subfield thickness.	No advantage: CST difference about -2 μm; VA difference about 0.4 letters; no serious related adverse events.	Independent DRCR Retina Network trial; different disease biology and device from AMD trials.

Abbreviations: AMD, age-related macular degeneration; BCVA, best-corrected visual acuity; DME, diabetic macular edema; FDA, United States Food and Drug Administration; OCT, optical coherence tomography; PBM, photobiomodulation.

**Table 4:** Representative PBM protocols and optical heterogeneity in the studies discussed.

Study/Model	Source and Wavelength(s)	Delivery Schedule	Endpoint Context	Interpretation
Methanol/formate retinal toxicity [34]	670 nm LED	Acute rescue protocol in toxic respiratory-chain inhibition model.	ERG and retinal/optic-nerve structural protection.	Mechanistically relevant to respiratory inhibition, but unlike chronic degeneration.
Light-induced retinal degeneration [35,36]	Red/NIR exposure, commonly 670 nm in related models.	Peri- or post-injury experimental exposure.	Photoreceptor survival, Müller-cell and microglial/macrophage activation.	Supports photo-oxidative rescue and anti-inflammatory signals, but model is acute.
Blue-light photoreceptor injury [37]	670 and 810 nm	Cell-culture exposure in primary murine photoreceptors.	Cell survival and respiratory-chain involvement.	Suggests mechanisms beyond Complex IV alone.
P23H retinitis pigmentosa model [38]	830 nm	Repeated PBM in inherited degeneration model.	Mitochondrial redox state, retinal morphology and function.	Important progressive-disease model but still one genotype/protocol.
Diabetic retinopathy model [40]	PBM protocol as reported in diabetic mouse study.	Long-term exposure during sustained metabolic disease.	Structural and functional lesions of diabetic retinopathy.	Clinically relevant chronic context, but systemic disease complicates translation.
Valeda AMD trials [42,43,44,45,48]	590, 660 and 850 nm	Nine sessions over 3–5 weeks; repeated series in several protocols.	BCVA, contrast sensitivity, drusen/OCT and safety outcomes.	Most standardized clinical evidence; device-specific and sponsor-concentrated.
Protocol AE in DME [49]	670 nm PBM eye patch	90 s twice daily for 4 months.	OCT central subfield thickness and VA.	Negative result underlines disease and device specificity.

**Table 5:** Reporting and interpretation parameters for retinal PBM studies.

Parameter	Why It Matters	Practical Comment
Wavelength and spectral bandwidth	Determine chromophore interaction, tissue penetration, and retinal exposure.	Distinguish single-wavelength red light from multiwavelength protocols such as those used in dry AMD trials [41,42,43,44,45].
Irradiance and fluence	Define the rate and total amount of energy delivered.	Similar fluence values may not produce identical effects if irradiance and exposure time differ [23,24].
Exposure duration and number of sessions	Influence biological adaptation and durability.	Retinal PBM is likely to require protocol-specific retreatment schedules rather than one universal regimen.
Continuous versus pulsed delivery	May affect mitochondrial, vascular, or neural responses.	Pulsing should be reported precisely and not treated as a minor device detail.
Timing of treatment	May interact with retinal metabolic and circadian biology.	Time-of-day effects remain hypothesis-generating for disease protocols and should be tested prospectively.
Ocular and disease status	Determines the biological substrate available for response.	Lens status, media opacity, pigmentation, retinal atrophy, and prior treatments may influence retinal light exposure and interpretation.
Safety monitoring	Essential because the retina is light-sensitive neural tissue.	OCT, fundus examination, functional testing, and adverse-event reporting should be built into protocols.

Abbreviations: AMD, age-related macular degeneration; OCT, optical coherence tomography; PBM, photobiomodulation.

## Data Availability

No new data were created or analyzed in this study.
